# Impact of COVID‐19 on Hospital Admissions for Children With Developmental Disadvantages: A Western Sydney Metropolitan Hospital Experience on Health Inequity

**DOI:** 10.1111/jpc.16798

**Published:** 2025-02-05

**Authors:** Lanxin Li, Habib Bhurawala, Anthony Liu

**Affiliations:** ^1^ Nepean Clinical School Faculty of Medicine and Health, The University of Sydney Kingswond New South Wales Australia; ^2^ Paediatric Department Nepean Hospital Kingswood New South Wales Australia; ^3^ Faculty of Medicine University of Notre Dame Australia Darlinghurst Sydney Australia

**Keywords:** autism spectrum disorder, COVID‐19, developmental disabilities, health inequities

## Abstract

**Aims:**

To investigate the impact of Sydney's COVID‐19 lockdown on children with developmental disadvantages and reflect on current clinical practice.

**Methods:**

Retrospective data was collected from a western Sydney metropolitan hospital's electronic database and patients. We reviewed all children with Autism Spectrum Disorder (ASD), Cerebral Palsy (CP) or Intellectual Disability (ID) admitted 36 months prior to and after lockdown policy being implemented.

**Results:**

The prevalence of hospital presentation was reduced during the lockdown period. The average length of stay (LoS) increased by approximately 1.5 folds. Comparatively, the average LoS of the general paediatric population increased minimally. Seizures, asthma, and upper respiratory tract infections were the three commonest issues for hospital admissions in both periods. COVID‐19 infection accounted for 4% of admissions in the lockdown period. Around 20.8% of patients with disadvantages were admitted with more than two major issues. None of these patients had concurrent COVID‐19 infection.

**Conclusions:**

Children with developmental disabilities experience greater disadvantages during lockdown. The likely reasons include inequity caused by increased barriers to healthcare service. The indirect benefit of reducing infection transmission during lockdown was not reflected in our patient group, contributing to the disease burden. Chronic diseases remain the most common causes of admissions in all periods, suggesting the essentiality of improving chronic disease management in future clinical practice.

**Summary**

- What is already known on this topic?
  ○ It is well established that paediatric patients with developmental disadvantages experience greater healthcare burdens compared to the general paediatric patient population.
  ○ Lockdown restrictions, such as maintaining social distancing and mandatory masking wearing, have indirect health benefits in preventing airborne disease transmission.
  ○ Barriers increase for patients seeking medical service during the lockdown period.
- What this paper adds?
  ○ Paediatric patients with disadvantages experience disproportionally greater health burdens during lockdown compared to the general paediatric population.
  ○ The indirect health benefits of the reduction of airborne diseases, such as URTI, were not reflected in paediatric patients with developmental disadvantages.
  ○ Regardless of the relatively increased barriers to entry to healthcare services, hospital presentations due to chronic diseases remain high during lockdown for patients with disadvantages.

## 1 Introduction

The COVID‐19 pandemic has caused a great health burden globally [1]. Nationwide strategies have been adopted in Australia to counteract the pandemic stress, including placing health restrictions in late March 2020 in each state [2]. To date, evidence of the impact of COVID‐19 and relevant healthcare restrictions are area‐specific and conducted in the general population composed of mostly adult patients, suggesting increased hospital length of stay [3], implicating the negative impact on patient health. Paediatric studies were more limited, with conflicting results. Some studies show that children experience fewer comorbidities compared to adults when exposed to COVID‐19, though the reasons remain unclear [4, 5].

Based on the most recent statistical report, Autism Spectrum Disorders (ASD) and Intellectual Disabilities (ID) account for the two most common developmental disadvantages in the paediatric population in Australia [6]. Several disease associations have been established with the developmental disadvantageous conditions. Asthma, one of the most common diseases causing hospitalisation in the paediatric population, has been found to be closely linked to ASD [7, 8]. Chronic gastrointestinal‐associated symptoms, including changes in bowel habits, stool consistency, abdominal pain, and coeliac disease, have also been demonstrated [9]. Other chronic disease associations including concerns regarding safety issues during in‐patient admissions have also been demonstrated [10, 11]. Overall, these patients have a more significant long‐standing healthcare burden. On the other hand, these patients also experience greater disadvantages in acute medicine settings. Patients with disadvantages present more frequently to the emergency department (ED) compared to the general paediatric populations, with most presentations as ambulatory care‐sensitive conditions [12, 13]. While physical health issues remain the main causative complaints to ED [14], psychiatric or behavioural issues also cause frequent admissions for children and adolescents with developmental disabilities [15]. The latter conditions are worth considering in the setting of pandemics, as mental health stress seems to spike during the period.

To date, Australian studies on the impact of COVID‐19 on children have been limited, and no study has focused on children with disadvantageous conditions. Globally, evidence suggests these patients experience increased family tension discontinuation of relevant therapies, and reduced mental health due to reduced healthcare access [16, 17]. While primary healthcare sectors have implemented strategies such as Telehealth to counteract the psychosocial stress for patients and ease the caring burden for their carers [18, 19], the interaction between patients, carers and tertiary healthcare facilities remains unclear.

We hypothesise that COVID‐19 has a more significant negative impact on children with developmental disadvantages. Therefore, we conducted a preliminary retrospective cross‐sectional study in a Western Sydney metropolitan hospital with a particular focus on patients with ASD, ID, and CP. We aim to demonstrate how these patient groups can be adversely impacted and highlight the areas for improvement in future practice.

## 2 Methods

### 2.1 Ethics Statement

This study has been reviewed by the local health district human research ethics committee under a low or negligible‐risk review pathway and deemed to meet the requirements of the National Statement on Ethical Conduct in Human Research (2007).

### 2.2 Participant Recruitment

Patient information was retrieved from the hospital's electronic medical records and the hospital's Department of Paediatrics' admission database. All data was de‐identified. The original collection of data was processed by department administration staff not involved in the medical treatment team, avoiding bias in patient selection. The pre‐COVID‐19 period was defined as 28th March 2017 to 27th March 2020, which is 3 years (36 months) before COVID‐19 lockdown. The lockdown period was defined as 28th March 2020 to 27th Mar 2023, accounting for 3 years (36 months) affected by COVID‐19 lockdown.

### 2.3 Exposure Group: Patients With Autistic Spectrum Disorder (ASD), Cerebral Palsy (CP), or Intellectual Disability (ID)

We defined patients with ASD, CP, or ID as those with developmental disadvantages. Patients with ASD were identified by searching admission notes. The following terms were used to identify patients with ASD: ‘autistic spectrum disorder’, ‘autism’, ‘autistic’, and ‘ASD’.

Patients with CP were identified by searching admission notes. The following terms were used to identify patients with CP: ‘cerebral palsy’, ‘CP’, ‘cerebral’, and ‘palsy’.

Patients with ID were identified by searching admission notes. The following terms were used to identify patients with ID: ‘intellectual disability’, ‘ID’, ‘intellectual’, and ‘disability’.

### 2.4 Outcome Measures and Data Collection

The following parameters were extracted from the electronic Medical Recording(eMR) of selected patients:

Hospital length of stay (LoS), weight and weight percentile using Growth Charts from Centers for Disease Control and Prevention (CDC) (2000) (Accessed 17th Oct 2023, https://www.cdc.gov/growthcharts/clinical_charts.html), and age.

LoS was compared between general paediatric populations in pre‐ and COVID‐19 lockdown periods, as well as between patients with disadvantages in the same periods. Then, the LoS of patients with disadvantages was compared with general paediatric populations in pre‐ and COVID‐19 lockdown periods, respectively. Weight percentiles were analysed in selected patient groups in pre‐ and COVID‐19 lockdown periods.

The main complaints causing hospital admissions were retrieved from the eMR. A total of 43 conditions were recorded in both the pre‐ and COVID‐19 lockdown periods.

### 2.5 Statistical Analysis

Statistical analysis was conducted using Microsoft Office Excel 2010 Data Analysis Toolpak and IBM SPSS Statistics for Windows, Version 28.0. Armonk, NY: IBM Corp. Released 2021. We used Independent‐Samples T Test and One‐way ANOVA for comparing continuous variables, with Levene's Test for homogeneity of variances. We used the Crosstabs function and Pearson Chi‐square tests for categorical variables and independence. We accepted *p* < 0.05 as statistically significant for all tests unless with additional comments otherwise.

## 3 Results

### 3.1 Overview of Hospital Admissions in Pre‐ and During COVID Lockdown

During the pre‐lockdown period, 9736 total admissions were recorded in the electronic medical records. Of these, 151 admissions were related to patients with developmental disadvantages (1.6% of overall paediatric admission). In contrast, 8253 total admissions were recorded during lockdown period. Of these, 69 admissions were related to patients with developmental disadvantages (0.8% of overall paediatric admission). The details of the prevalence of selected patient groups admitted in the pre‐ and lockdown periods were demonstrated in Table 1. The proportion of each specific condition, ASD, CP, and ID was shown in Table 2.

**TABLE 1:** Prevalence of patients with developmental disadvantages in pre‐ and lockdown periods.

		COVID lockdown status*	
		Pre‐lockdown	Lockdown
Patients with disadvantages	% within COVID lockdown status	01.6% _a_	00.8% _b_
General paediatric patients	% within COVID lockdown status	98.4% _a_	99.2% _b_
Total	% within COVID lockdown status	100.0%	100.0%

* Each subscript letter denotes a subset of COVID lockdown status categories whose column proportions do not differ significantly from each other at the 0.05 level.

**TABLE 2:** Prevalence of patients with ASD, CP or ID out of all patients with developmental disadvantages in pre‐ and lockdown period.

		COVID lockdown status*	
		Pre‐lockdown	Lockdown
ASD	% within COVID lockdown status	80.8% _a_	69.6% _a_
CP	% within COVID lockdown status	12.6% _a_	26.1% _b_
ID	% within COVID lockdown status	6.6% _a_	4.3% _a_
Total	% within COVID lockdown status	100.0%	100.0%

* Each subscript letter denotes a subset of COVID lockdown status categories whose column proportions do not differ significantly from each other at the 0.05 level.

The proportion of selected patients admitted to the hospital has decreased in the period affected by COVID (*p* < 0.001). In contrast, CP patients formed a greater proportion within the patient population with specific conditions (*p* < 0.05).

### 3.2 Average LoS in Pre‐ and Lockdown Period

From 28th Mar 2017 to 27th Mar 2020, the average LoS for all paediatric patients admitted was 1.85 days (±0.071, 95% CI [1.779–1.921]). For patients with ASD, CP, and ID, the average LoS was 1.95 days (±0.493, 95% CI [1.457–2.443]) during the same period. From 28th Mar 2020 to 27th Mar 2023, the average length of stay for all paediatric patients was 2.52 days (±0.100, 95% CI [2.420–2.620]). For patients with ASD, CP, and ID admitted during the same period, the average length of stay was 4.87 days (±3.204, 95% CI [1.666–8.074]).

### 3.3 Weight of Patients With Developmental Disadvantages at Admission in Pre‐ and Lockdown Period

Within 116 valid entries during 3 years pre‐COVID‐19 lockdown, the average percentile of weight for patients with disadvantages admitted was 58.5 (±0.133, 95% CI [52.367–64.633]). Within 66 valid entries during 3 years affected by COVID‐19 lockdown, the average weight percentile for patients with disadvantages was 55.5 (±9.168, 95% CI [46.332–64.668]).

### 3.4 Main Issues Causing Hospital Admissions for Selected Patients in Pre‐ and Lockdown Period

**FIGURE 1:**
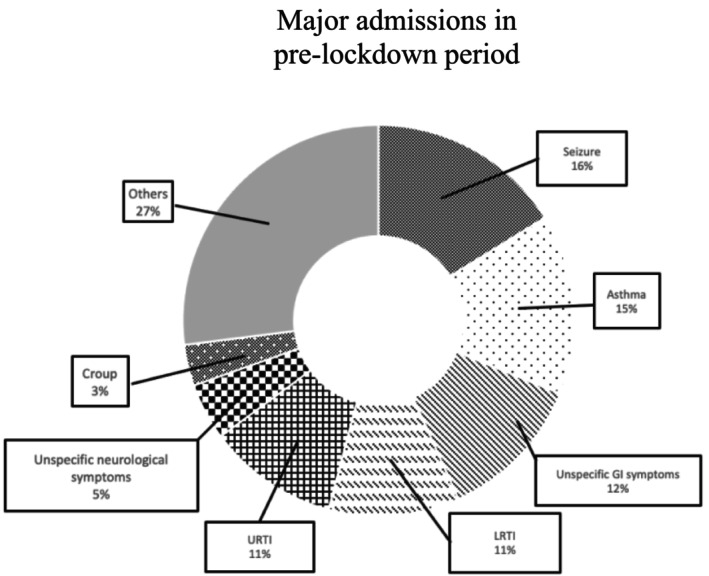
Top seven major issues causing hospital admissions for patients with disadvantages in the pre‐lockdown period. Seizures are the commonest, with 24 admissions, occupying 16% of total admissions. GI, gastrointestinal; LRTI, lower respiratory tract infection; URTI, upper respiratory tract infection.

In pre‐lockdown period, 38 out of 148 admissions were recorded as having at least two main issues at admission (25.7%). Top seven commonest issues causing hospital admissions are demonstrated in Figure 1 below.

**FIGURE 2:**
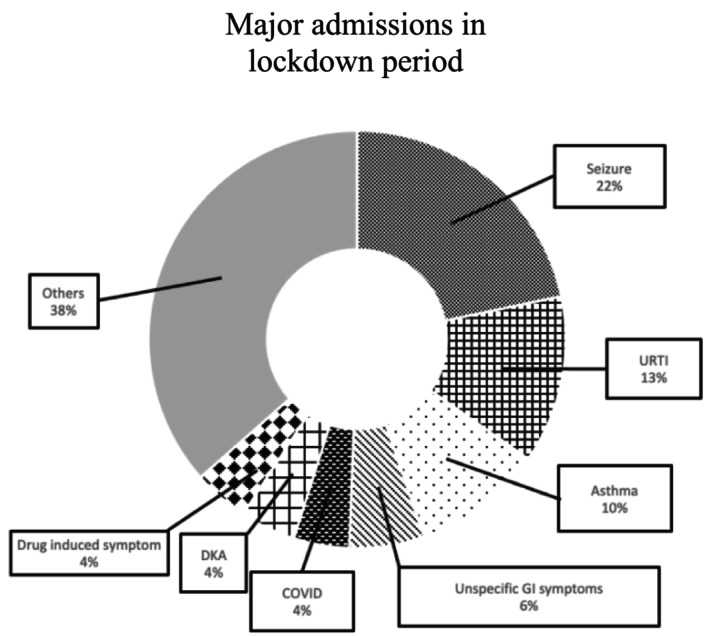
Top seven major issues causing hospital admissions for patients with disadvantages in lockdown period. Seizures are the commonest, with 14 admissions, occupying 22% of total admissions. COVID refers to admissions directly caused by COVID‐19 infections. Drug‐related symptoms refer to drug overdose or misuse. DKA, diabetic ketoacidosis; GI, gastrointestinal; URTI, upper respiratory tract infection.

During lockdown period, 14 out of 69 admissions were recorded as having at least two main complaints at admission (20.3%). Again, top seven commonest issues causing hospital admissions are demonstrated in Figure 2. below. COVID accounts for 4% of total admissions, ranked as one of the top seven most common issues. The same issues were coded with the same colour in Figures 1 and 2.

**FIGURE 3:**
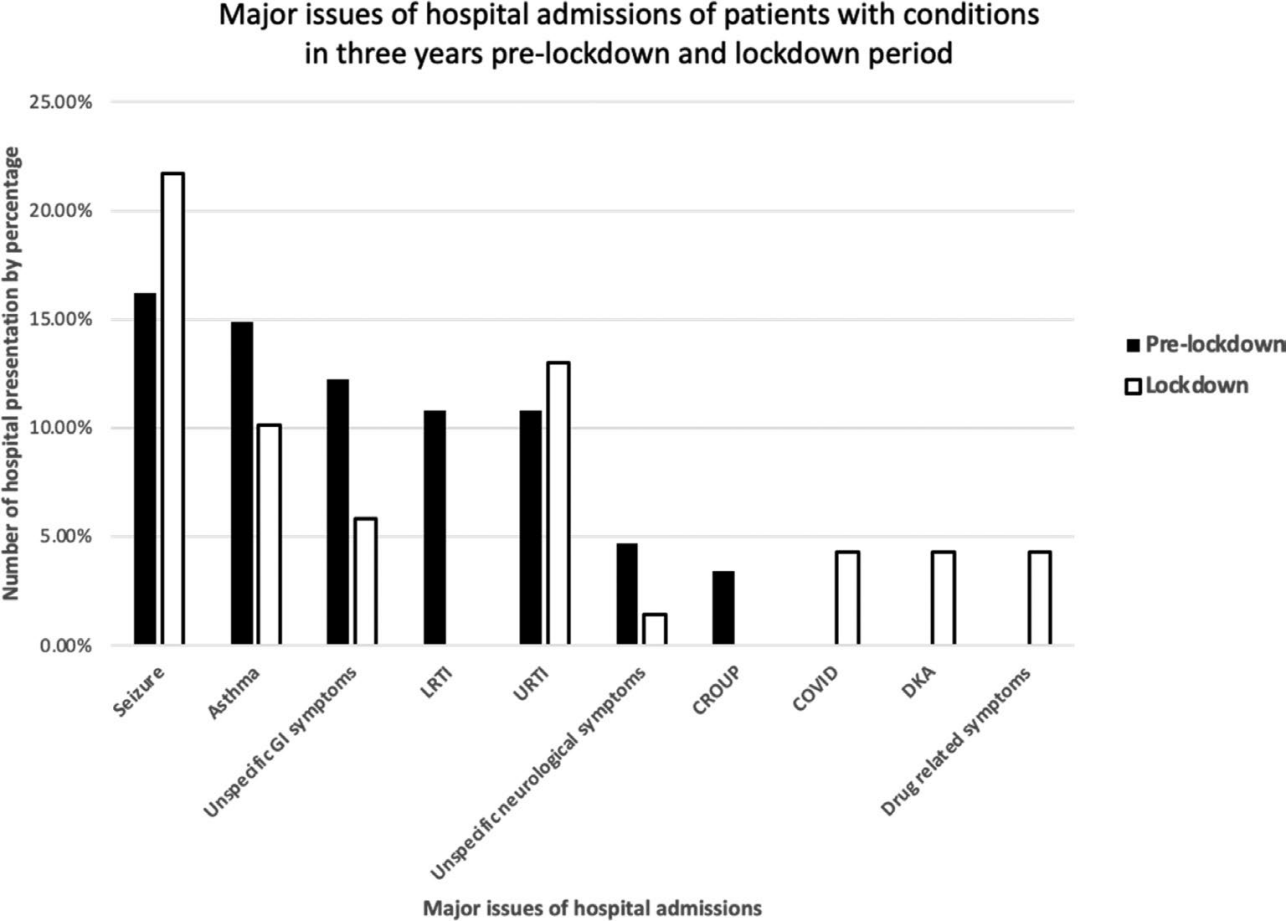
Comparison of top seven major issues causing hospital admissions in pre‐ and lockdown periods. A total of 10 types of issues were recorded. Seizures were the commonest in both periods. No admission in pre‐lockdown period was related to COVID, DKA or drug‐related symptoms. No admission during the lockdown period was related to LRTI or CROUP.

The major issues of hospital admissions for patients with developmental disadvantages were further demonstrated in Figure 3, and the pre‐lockdown and lockdown groups did not overlap completely. Admissions related LRTI and CROUP were not recorded during lockdown period, whereas admissions related to COVID, DKA, and drug‐related symptoms were not recorded in pre‐lockdown period.

## 4 Discussion

We are the first Australian study investigating the impact of COVID‐19 and lockdown on paediatric hospital admissions with a focus on special patient groups affected by developmental disadvantages. Our results demonstrated hospital admissions of children with developmental disadvantages were reduced during the lockdown period. However, this is unnecessarily corresponding to the extent of disease burden, rather due to the lockdown policies. The launch of various social restrictions may limit patients with disadvantages and their carers seeking medical services, including presenting to hospitals. The delivery of essential long‐term health services might be interrupted [20]. Indeed, by further investigating the admission notes, recurrent short‐term same‐day admissions existed for patients requiring regular procedures such as iron infusions, whereas such recording was not found in the post‐lockdown period. Moreover, in the post‐lockdown period, less patients were admitted for overnight observations from the emergency department. While this could suggest more patients were directly discharged from the emergency department without being admitted, the safety post‐discharge was not guaranteed. Furthermore, our study suggests patients with disadvantages stayed on average 1.5‐fold longer in the hospital per admission during the lockdown period. In comparison, the length of stay for the general paediatric population was only more than 0.4 times longer. Together, this implies for those patients with disadvantages admitted during the lockdown period, the disease is more severe, proving our hypothesis of these patients experiencing disproportional disadvantages in lockdown.

We then investigated the main medical issues of hospital presentations for the selected patient groups. Surprisingly, unlike what we expected, COVID‐19 infection only occupies 4% of the total presentations amongst patients with disadvantages without requiring escalation of care, such as intensive care unit involvement. This was no noticeable difference compared to the rest of the general paediatric population worldwide [21].

Unlike other studies on the general paediatric population suggesting a down‐trending pattern in respiratory infectious disease [22, 23], URTI has remained one of the top four most common presentations in both pre‐lockdown and lockdown periods for patients with disadvantages. While public health orders such as mandatory mask‐wearing and appropriate social distancing may contribute to the indirect effect of limiting transmission of the respiratory virus amongst the general paediatric population, the seemingly positive impact of lockdown was not reflected in our study. A qualitative approach was adopted for further investigations. All ASD patients were classified at least level 2 with pre‐existing recordings of high‐risk carer's stress. While compliance with the infectious disease control method remains an important method of limiting disease transmissions, factors such as worsened family tension and increased socioeconomic stress were identified, likely explaining the discrepancies in the observations between the patients with developmental disadvantages and general paediatric populations. Interestingly, admissions for Lower Respiratory Tract infections (LRTI), especially pneumonia, have been significantly reduced. Indeed, there is no record related to LRTI. While the reasons behind the discrepancy remain unclear, these findings may warrant further studies with the expansion of patient group size.

The pattern of the presenting complaints of hospital admissions has also provided us with some implications for managing chronic diseases for patients with developmental disadvantages. In our selected patient groups, seizure and asthma exacerbation are ranked as the two most common presentations in both periods, implicating chronic diseases contributing to disease burden during pandemics. While it might imply the barriers discussed above limiting patients with ambulatory care‐sensitive conditions presenting to the hospital, patients with chronic conditions still experience significant burdens and require medical attention. In addition, this pattern seemed to be more clinically significant in patients with disadvantages, as other evidence was seen in a previous study suggesting a reduction of hospital admission due to chronic diseases in the general paediatric population during the lockdown [24]. Moreover, some of the presentations only related to the 3‐year lockdown period included diabetic ketoacidosis (DKA) and drug‐related issues. By investigating these patients' recordings, three of them were indirectly related to the pandemic, as two were due to missed education sessions due to the carer contracting COVID‐19, and one was caused by a carer in quarantine, resulting in patient mis‐ingesting supra‐therapeutic anti‐psychotics. Unfortunately, no qualitative evidence has been demonstrated in patients' records suggesting additional measures addressing the disruption of medical services for these patients. Additionally, with a similar pattern of admission presentations, the increase in approximate two‐fold LoS in the lockdown period again indirectly proves the hypothesis of the disproportional disadvantage of the health burden experienced by the patients with disadvantages.

Certainly, there are areas warrant further evaluation. Our results are more applicable to the patient groups growing up in western Sydney metropolitan regions. Future studies can consider the socioeconomics cultural background in analysing the relevant outcomes. Considering this factor, our patient population only occupies 2.4% of the total paediatric patient population, much less than the recorded prevalence Australia‐wide [6], implicating a likely underestimation of the patient within a disadvantaged population. The relatively small sample size of children with developmental disadvantages warrants caution in drawing definitive conclusions. Furthermore, the nature of the retrospective design and the reliance on hospital records may limit the generalisability of the findings. Future research should expand the patient population and incorporate qualitative methods to further explore the psychosocial impacts of the pandemic on families of children with developmental disadvantages. This would help better understand the long‐term consequences of COVID‐19 disruptions on this vulnerable group.

## 5 Conclusion

To our knowledge, this is the first Australian study investigating the impact of COVID‐19 on hospital admissions of paediatric patients with developmental disadvantages. Our study highlights the disproportionate impact of the COVID‐19 pandemic on children with developmental disadvantages, specifically those with ASD, ID, and CP. These children experienced increased disruptions to healthcare services, including longer hospital stays and reduced access to routine medical care. Despite the low frequency of COVID‐19‐related admissions, the consistent severity of their chronic illnesses, compounded by the pandemic's indirect effects, contributed to a greater healthcare burden. Our findings suggest that future pandemic preparedness plans should address the unique needs of children with developmental disabilities to mitigate disruptions in care to ensure that vulnerable populations continue to receive necessary services during times of crisis. While our study provides valuable insights, further research with a larger sample size and qualitative methods is needed to explore the long‐term psychosocial impacts on families and the ongoing healthcare needs of children with developmental disadvantages.

## Ethics Statement

This study was approved under code 2022/ETH02033 by the Nepean Blue Mountains Local Health District Human Research Ethics Committee.

## Consent

A waiver of consent was approved with review as a low or negligible‐risk review pathway.

## Conflicts of Interest

The authors declare no conflicts of interest.

## References

[1] World Health Organization (WHO) , “COVID‐19 Weekly Epidemiological Update,” 2021.

[2] Statistics ABo , “Impact of Lockdowns on Household Consumption ‐ Insights From Alternative Data Sources Internet: Australian Bureau of Statistics,” last modified December 1, 2021, https://www.abs.gov.au/articles/impact‐lockdowns‐household‐consumption‐insights‐alternative‐data‐sources.

[3] S. Chia , J. Xia , Y. H. Kwan , et al., “Evaluating the Association of COVID‐19 Restrictions on Discharge Planning and Post‐Discharge Outcomes in the Community Hospital and Singapore Regional Health System,” Frontiers in Health Services 3 (2023): 1147698. 37744642 10.3389/frhs.2023.1147698 PMC10513784

[4] S. Tosif , L. F. Ibrahim , R. Hughes , et al., “Characteristics and Outcomes of SARS‐CoV‐2 Infection in Victorian Children at a Tertiary Paediatric Hospital,” Journal of Paediatrics and Child Health 58, no. 4 (2022): 618–623. 34693586 10.1111/jpc.15786 PMC8662161

[5] T. Beaney , A. L. Neves , A. Alboksmaty , et al., “Trends and Associated Factors for Covid‐19 Hospitalisation and Fatality Risk in 2.3 Million Adults in England,” Nature Communications 13, no. 1 (2022): 2356. 10.1038/s41467-022-29880-7 PMC9054846 35487905

[6] Health AIo, Welfare , Autism in Australia (Canberra: AIHW, 2017).

[7] M.‐H. Chen , T.‐P. Su , Y.‐S. Chen , et al., “Comorbidity of Allergic and Autoimmune Diseases in Patients With Autism Spectrum Disorder: A Nationwide Population‐Based Study,” Research in Autism Spectrum Disorder 7, no. 2 (2013): 205–212.

[8] Z. Zheng , L. Zhang , T. Zhu , J. Huang , Y. Qu , and D. Mu , “Association Between Asthma and Autism Spectrum Disorder: A Meta‐Analysis,” PLoS One 11, no. 6 (2016): e0156662. 27257919 10.1371/journal.pone.0156662 PMC4892578

[9] G. Leader and A. Mannion , “Gastrointestinal Disorders,” in Comorbid Conditions Among Children With Autism Spectrum Disorders, ed. J. L. Matson (Cham: Springer International Publishing, 2016), 257–281.

[10] A. K. Koyama , E. H. Koumans , K. Sircar , et al., “Severe Outcomes, Readmission, and Length of Stay Among COVID‐19 Patients With Intellectual and Developmental Disabilities,” International Journal of Infectious Diseases 116 (2022): 328–330. 35077878 10.1016/j.ijid.2022.01.038 PMC8783540

[11] L. Mimmo , R. Harrison , J. Travaglia , N. Hu , and S. Woolfenden , “Inequities in Quality and Safety Outcomes for Hospitalized Children With Intellectual Disability,” Developmental Medicine and Child Neurology 64, no. 3 (2022): 314–322. 34562021 10.1111/dmcn.15066 PMC9293445

[12] D. Iannuzzi , M. Hall , N. M. Oreskovic , et al., “Emergency Department Utilization of Adolescents and Young Adults With Autism Spectrum Disorder,” Journal of Autism and Developmental Disorders 52, no. 2 (2022): 617–622. 33751374 10.1007/s10803-021-04969-y

[13] P. S. Carbone , P. C. Young , G. J. Stoddard , J. Wilkes , and L. Trasande , “A Comparison of Ambulatory Care Sensitive Hospitalizations Among Children With and Without Autism Spectrum Disorder,” Academic Pediatrics 15, no. 6 (2015): 626–635. 26547543 10.1016/j.acap.2015.07.006

[14] M. S. Mannenbach , R. L. Passe , K. K. Lovik , et al., “Caring for Children With Autism in an Emergency Department Setting,” Pediatric Emergency Care 37, no. 12 (2021): e977–e980. 33170575 10.1097/PEC.0000000000001844

[15] J. E. Rast , A. M. Roux , S. J. Fernandes , V. D'Silva , and L. L. Shea , “Hospital Inpatient Stays for Autistic Youth and Youth With Other Disabilities,” Pediatrics 149, no. S4 (2022): e2020049437R. 10.1542/peds.2020-049437R 35363287

[16] C. Isensee , B. Schmid , P. B. Marschik , D. Zhang , and L. Poustka , “Impact of COVID‐19 Pandemic on Families Living With Autism: An Online Survey,” Research in Developmental Disabilities 129 (2022): 104307. 35908370 10.1016/j.ridd.2022.104307 PMC9271458

[17] J. S. Sanders , R. L. I. Pillai , R. Sturley , et al., “Impact of the COVID‐19 Pandemic on the Behavioral Health of People With Intellectual and Developmental Disabilities,” Psychiatric Services 73 (2022): 1389–1392. 35734865 10.1176/appi.ps.202100524

[18] E. A. Craig , K. Dounavi , and J. Ferguson , “Effectiveness of a Brief Functional Analysis and Functional Communication Training Conducted Through Telehealth,” Journal of Developmental and Physical Disabilities 35 (2022): 227–246. 35967272 10.1007/s10882-022-09857-6 PMC9358095

[19] J. Brian , A. Solish , E. Dowds , et al., “Going Mobile‐Increasing the Reach of Parent‐Mediated Intervention for Toddlers With ASD via Group‐Based and Virtual Delivery,” Journal of Autism and Developmental Disorders 52 (2022): 5207–5220. 35608785 10.1007/s10803-022-05554-7 PMC9128315

[20] R. Baweja , S. L. Brown , E. M. Edwards , and M. J. Murray , “COVID‐19 Pandemic and Impact on Patients With Autism Spectrum Disorder,” Journal of Autism and Developmental Disorders 52, no. 1 (2022): 473–482. 33689088 10.1007/s10803-021-04950-9 PMC7943706

[21] NSW Health , Report COVID‐19 Critical Intelligence Unit: Paediatrics and COVID‐19 – Reporting, Rates and Differences (St Leonards: NSW Health, 2022).

[22] S. Kadambari , R. Goldacre , E. Morris , M. J. Goldacre , and A. J. Pollard , “Indirect Effects of the Covid‐19 Pandemic on Childhood Infection in England: Population Based Observational Study,” BMJ 376 (2022): e067519. 35022215 10.1136/bmj-2021-067519 PMC8753487

[23] M. D. Kruizinga , D. Peeters , M. van Veen , et al., “The Impact of Lockdown on Pediatric ED Visits and Hospital Admissions During the COVID19 Pandemic: A Multicenter Analysis and Review of the Literature,” European Journal of Pediatrics 180, no. 7 (2021): 2271–2279. 33723971 10.1007/s00431-021-04015-0 PMC7959585

[24] N. Hu , N. Nassar , J. Shrapnel , et al., “The Impact of the COVID‐19 Pandemic on Paediatric Health Service Use Within One Year After the First Pandemic Outbreak in New South Wales Australia a Time Series Analysis,” Lancet Regional Health – Western Pacific 19 (2022): 100311. 34746898 10.1016/j.lanwpc.2021.100311 PMC8564784

